# Tool Comparison for Detecting Tumour Cells in Endometrial Cancer via Single‐Cell Copy Number Variations Analysis

**DOI:** 10.1111/jcmm.70932

**Published:** 2025-10-29

**Authors:** Erica Dugo, Francesco Piva, Matteo Giulietti, Luca Giannella, Andrea Ciavattini

**Affiliations:** ^1^ Department of Specialistic Clinical and Odontostomatological Sciences Polytechnic University of Marche Ancona Italy; ^2^ Gynecologic Section, Woman's Health Sciences Department Polytechnic University of Marche Ancona Italy

**Keywords:** biomarkers, copy number variations (CNVs), endometrial cancer (EC), epithelial cells, single‐cell RNA transcriptome

## Abstract

Copy number variations (CNVs) are considered a hallmark of cancer and their inference from high‐resolution single‐cell transcriptome (scRNA‐seq) analyses may offer great opportunities for the study of tumor heterogeneity. We compared the results of four major tools (SCEVAN, CopyKAT, InferCNV and sciCNV) that use inferred CNVs to predict endometrial cancer (EC) cells, in order to assess their reliability and offer useful suggestions to researchers to improve the accuracy of their predictions. In this study, we identified EC cells from publicly available scRNA‐seq data using well‐established EC biomarkers reported in the literature. SCEVAN and CopyKAT tools have moderate sensitivity, but significantly overestimate the true number of true EC tumour cells. However, a comparative analysis of the different tumour subclones revealed that a lower number of false positives can be obtained by selecting only those that contain a high percentage of epithelial cells. In contrast, InferCNV and sciCNV do not directly predict tumour cells, but rather infer CNVs and compute CNV scores. However, the score distribution curves of the CNV scores did not clearly distinguish between malignant and non‐malignant cell populations, and therefore we were unable to evaluate the performance of either software. We highlight the lack of agreement between the tools and also towards the expected results. Our findings suggest exercising caution in the automated use of these tools. Until more accurate algorithms become available, we recommend filtering predictions ensuring that the necessary but not sufficient condition that the predicted tumour cells are at least epithelial is met.

**Abbreviations**
aCGH: array comparative genomic hybridisation
CNVs: copy number variations
CopyKAT: copy number karyotyping of aneuploid tumours
EC: endometrial cancer
EEC: endometrial endometrioid carcinoma
FIGO: International Federation of Gynaecology and Obstetrics
FISH: fluorescence in situ hybridization
GEO: gene expression omnibus
InferCNV: inferring copy number variations
RT‐qPCR: quantitative reverse transcription PCR
SCEVAN: single cell variational aneuploidy analysis
sciCNV: inferring single‐cell CNV
scRNA‐seq: single cell RNA sequencing
SNP: single nucleotide polymorphism array
TP: true positive

## 1 Introduction

Endometrial cancer (EC) has become increasingly prevalent in recent years, particularly in developed countries, leading to a rise in both morbidity and mortality rates. In fact, according to the American Cancer Statistics, approximately 69,000 new cases were diagnosed in the United States in 2025 [1]. Several factors are known to contribute to the risk of developing EC, including early menarche, nulliparity, obesity, elevated levels of circulating oestrogen, late menopause, and familial history of endometrial cancer. Histological analysis classifies the disease by type and grade, while clinical examination, which can be supplemented by histological and molecular information data, determines its stage. The WHO recognised a mutation‐spectrum‐based molecular classification in 2020 [2], and the International Federation of Gynaecology and Obstetrics (FIGO) staging system was updated in 2023 to incorporate this molecular classification [3].

EC is widely recognised as a tumour with significant inter‐ and intra‐patient heterogeneity, which can be due to the presence of a different spectrum of mutations and copy number variations (CNVs). CNVs are segments of DNA that are duplicated or deleted to varying degrees across different cells, and their formation is primarily associated with DNA repair and replication mechanisms [4, 5].

These variations are common in many diseases, including cancers where their increased frequency often correlates with tumour onset and progression. The number of CNVs and type of genes involved, such as gains in proto‐oncogenes and losses in tumour suppressor genes, could be used to identify tumour cells [PMID: 41009333 PMID: 40898423]. For example, copy number gains of oncogenes such as MYC, ERBB2, and KRAS have been frequently observed in EC. Moreover, it has been observed that a higher number of CNVs present in histotype 2 EC, compared to type 1 EC, are correlated with poorly differentiated and more aggressive cells, resulting in more unfavourable prognoses [6]. This demonstrates that the number of CNVs can also be related to prognosis, but this association could be more accurately derived if the role of the gained or lost genes is also taken into account. In addition, when CNVs that influence the expression of genes known to be associated with drug resistance, histological grade or survival are identified, a more detailed prognostic significance can be attributed to their presence.

There are specific techniques for evaluating the presence of CNVs (e.g., aCGH, FISH, SNP array, etc.) and precise gene expression (e.g., RT‐qPCR) [7], but recently researchers have chosen to perform high‐resolution transcriptomic experiments to obtain functional insights in pathology. As these transcriptomic data are more often publicly available, they can also be used to infer information about CNVs.

High‐resolution transcriptomics include single cell RNA sequencing (scRNA‐seq), a novel technique that is able to evaluate the transcriptome of each individual cell in a sample and hence provide an understanding of the heterogeneity and complexity existing inside a tumour [8]. scRNA‐seq data are subjected to a quality control step to eliminate dead or damaged cells, cells with a high mitochondrial gene content, and sometimes even cells that correspond to doublets or multiplets. Clustering is then performed to group cells with similar expression profiles and are therefore of the same cell type and share similar functions.

An important step in scRNA‐seq data analysis is the attribution of a cell type to each cell based on its gene expression profile, a process known as cell annotation. This can be achieved by evaluating the expression of biomarkers characteristic of specific cell types [9]. Cell annotation also involves the identification of tumour cells, but only a few software are capable of this, and none are specific to EC. Most of these methods rely primarily on transcriptomic signatures, although some also incorporate CNV information [10, 11, 12, 13]. Moreover, different studies often propose distinct signatures for tumour cell identification [14].

CNV inference tools can only generate predictions because they are forced to derive genomic information from transcriptomic data. Several software have been developed for this purpose, including CopyKAT [15], SCEVAN [16], InferCNV [17], and sciCNV [18].

However, the results from these analyses are not fully reliable, due to the still limited sequencing depth [19] and our incomplete understanding of individual gene functions and their interactions.

Consequently, accurate identification of tumour cells from scRNA‐seq data in EC remains an unmet challenge, despite being crucial for downstream analyses.

In this study we compared the performance of different CNV‐based methods for identifying EC tumor cells from scRNA‐seq data, with the goal of evaluating their reliability and providing guidance to improve prediction accuracy.

## 2 Materials and Methods

### 2.1 Source of Single‐Cell RNA Sequencing Data

We analysed raw data files deposited in the GSE173682 dataset of the Gene Expression Omnibus (GEO) database (https://www.ncbi.nlm.nih.gov/geo/). We analysed the single‐cell transcriptomes of 5 patients diagnosed with EC who had not received adjuvant treatment and had EC with stage IA, endometrioid histology (EEC) and tumour grades ranging from 1 to 3. Their ages ranged from 49 to 70 years [20]. In addition, we analysed raw scRNA‐seq data of three healthy women aged 29–35 years as controls (GSE183837 dataset in the GEO database). Endometrial biopsies had been performed 5 days after ovulation [21]. The clinical characteristics of women with EEC and healthy women are shown in Table S1.

### 2.2 Quality Control, Normalisation, Feature Selection, and Dimension Reduction

The scRNA‐seq data was already pre‐processed by 10× Genomics' Cell Ranger software. We adopted Trailmarker (https://www.parsebiosciences.com/data‐analysis/) for all filtering, data quality control, normalisation and clustering operations. Quality control was performed on each gene expression matrix using the default parameters of the tool. Normalisation was achieved through the utilisation of the ‘LogNormalize’ function. To reduce the batch effect, scRNA‐seq data integration was performed with the ‘Harmony’ default function. Gene features were selected from the 2000 Highly Variable Genes (HVGs) using the ‘variance stabilizing transformation’ (vst) method for UMI (Unique Molecular Identifiers) count data. We used the Uniform Manifold Approximation and Projection (UMAP) as a method of dimensionality reduction and visualisation. The Louvain Clustering method was used at a different resolution for each patient to obtain clusters clearly distinguishable by gene expression, as shown by the heatmap. The resolution setting was 1.0 for patients 1, 2 and 5, 0.8 for patient 3, 0.7 for patient 4, 0.8 for control 1, 0.4 for control 2, and 0.6 for control 3.

### 2.3 Cell Type Annotation

Annotation of the cell types was done using the SingleR tool (version 2.2.0) [22], with HumanPrimaryCellAtlasData in the celldex package as the reference datasets.

### 2.4 Identification of EC Biomarkers and EC Tumour Cells

EC cancer cells were identified using biomarkers compiled from previously published studies and the Human Protein Atlas database (Table S2). In particular, we selected PubMed studies that had analysed EC genes with high expression or expressed exclusively in human EC since 2005. Most of these studies had been conducted on biopsy samples and on women who had not received any adjuvant treatment. We used Trailmarker to plot the level of gene expression and the percentage of cells expressing each biomarker previously retrieved (Table S2) in each cell cluster of each patient. Clusters containing cells expressing at least 40% of the EC biomarkers, at least from 80% of cells of the cluster or average expression levels higher than 1 were considered cancerous.

### 2.5 Inference of Copy Number Variations

To infer CNVs from scRNA‐seq data, we employed four computational tools: SCEVAN, CopyKAT, InferCNV, and sciCNV. These tools differ in their underlying algorithms, normalisation strategies, and approaches to CNV segmentation and classification (Supporting Information Methods and Table S3).

#### 2.5.1 SCEVAN

SCEVAN tool infers CNVs and uses them to automatically detect malignant and non‐malignant cells through a joint segmentation algorithm. To predict tumour cells by SCEVAN (version 1.0.1), we used the following parameters: SUBCLONES = TRUE since we were interested in analysing the clonal structure; ClonalCN = TRUE to obtain the profile of copy number variations for each tumour clone predicted by the program; the value of the segmentation parameter used is 0.5 (beta_vega); the value of the segmentation parameter (beta_vega) used is the default value (0.5) and a higher beta corresponds to coarser segmentation.

#### 2.5.2 CopyKAT

CopyKAT tool has the ability to automatically distinguish aneuploid malignant cells from diploid non‐malignant cells by inferring CNVs with an average resolution of 5 Mb. The identification of malignant cells based on the CNVs predicted by CopyKAT (version 1.1.0) was carried out using the following parameters: ngene.chr = 5 (requiring at least 5 genes per chromosome for cell filtering), win.size = 25 (defining 25 genes as the minimum window size for segmentation), KS.cut = 0.2 (segmentation parameter), distance = 'Euclidean' (clustering method utilised), norm.cell.names = ‘’, output.seg = ‘FALSE’, plot.genes = ‘TRUE’, genome = ‘hg20’.

#### 2.5.3 InferCNV

InferCNV tool employs a comparative approach by analysing gene expression intensity relative to the chromosomal position of reference ‘normal’ cells. CNV inference is performed by comparing the expression intensity of genes of the samples to be tested versus those of ‘normal’ cells. The immune cells used as reference cells were identified for each sample by SingleR. The cut‐off value was set at 0.1, as indicated in the InferCNV user guide for data from 10X Genomics platform. For every analyzed genomic window, InferCNV assigns a score ranging from 0 to 2, where values below 1 indicate copy number losses and values above 1 indicate copy number gains. To compare the overall CNV score in each cell, we first centred these values around 0 and then calculated their squared sum, so that deletions would have the same weight as amplifications, in order to obtain a CNV score for each cell. This method is commonly used to process raw InferCNV data in order to infer tumor cells [23, 24].

#### 2.5.4 sciCNV

sciCNV tool requires the input of a subset of normal cells to be used as the reference for inferring CNVs. CNV analysis with the sciCNV tool (version 0.99.73) was conducted using immune cells as ‘Reference’ (defined through SingleR) and all other cells as ‘Test’. The analysis was performed using the default parameters, that is, the number of most highly expressed genes in each cell used for RTAM normalisation was 250, the sharpness (resolution) of the analysis for the prediction of the preliminary CNVs in the ‘Test’ and ‘Reference’ cells was 1.0. After scaling, the data were subjected to noise filtering with a threshold of 0.2. A CNV score was then calculated for each Test and Reference cell using the formula described in the script CNV_score.R of sciCNV.

## 3 Results

### 3.1 Identification of Tumour Cells in Endometrial Cancer Through the Expression of Biomarkers

The identification of tumour cells in EC samples can be achieved by analysing the expression of biomarkers. For this purpose, we retrieved different EC markers proposed in the literature to compare them and evaluate if information about their use (e.g., which markers are necessary and sufficient) is known (Table S2).

The EC tumour biomarkers provided in Table S2 do not provide a precise identikit of tumour cells since the markers which are necessary, their potential interchangeability, and the minimum number required are not known. Additionally, there is no established formula that weighs the presence or absence of each marker in each cell to determine a final score or threshold for identifying cancerous cells.

**FIGURE 1:**
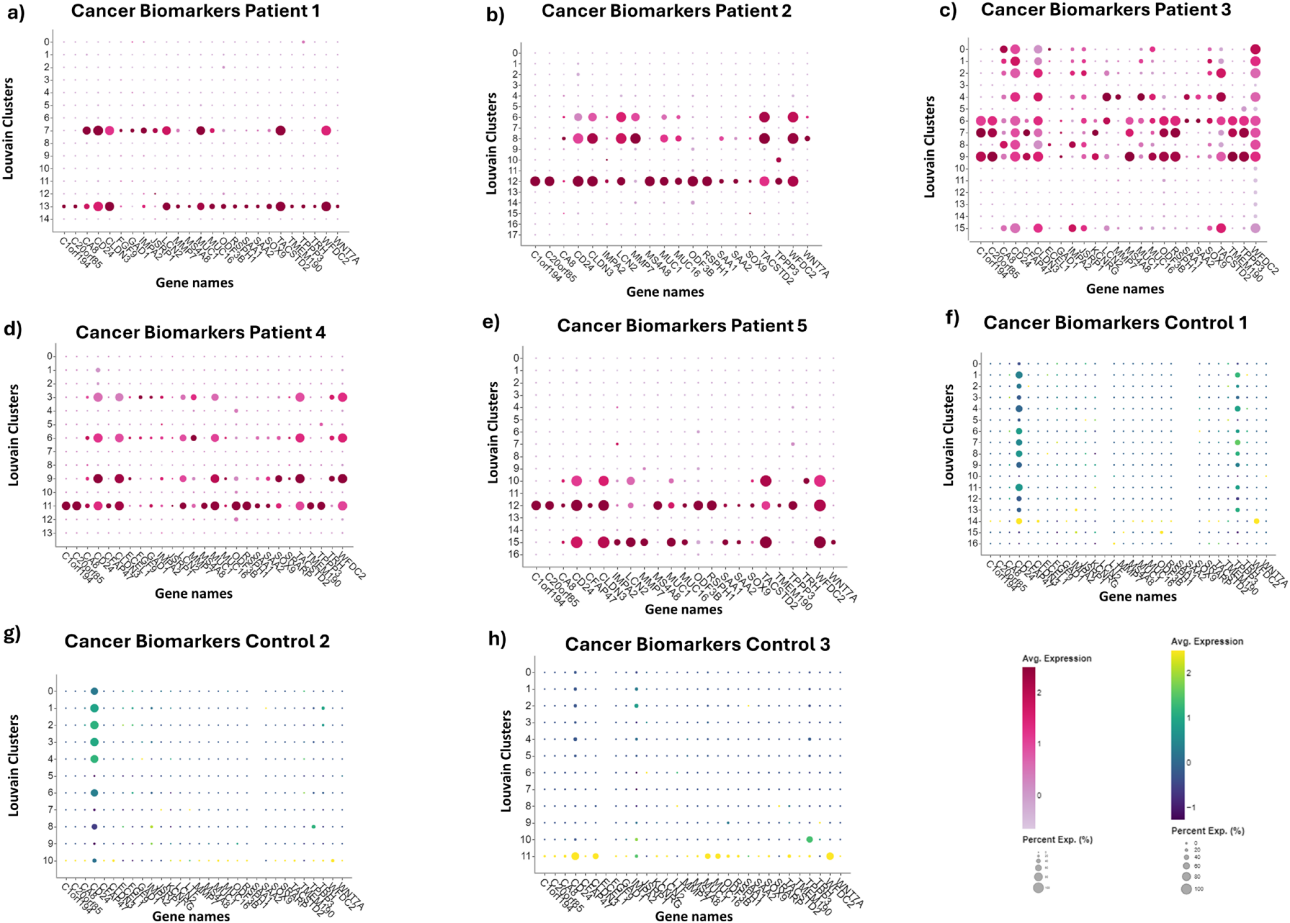
The dotplot of EC biomarker genes in tumour and control samples was generated by Trailmarker tool. The size of the plot shows the percentage of cells expressing a specific gene within the cluster, while the colour represents the average expression level across all cells within a cluster. (a–e) dot plot of tumour biomarker expression in patients 1, 2, 3, 4 and 5 respectively. (f–h) dot plot of tumour biomarker expression in controls 1, 2, and 3 respectively.

We verified the expression of these EC biomarkers in the analysed datasets, thus identifying specific clusters as cancerous (Figure 1a–e). In particular, in patient 1 two EC clusters with similar biomarker expression profiles were identified: 7 and 13. In patient 2 three clusters were identified: 6 and 8 having similar expression of biomarkers, and 12. In patient 3, nine EC clusters were identified which can be further grouped into two: clusters 0, 1, 2, 4, 8, 15, and clusters 6, 7, 9. In patient 4 there are four tumoral clusters of which clusters 3, 6 and 9 present similar expression of biomarkers and cluster 11 is characterised by a few more biomarkers. The three tumour clusters in patient 5 comprise two clusters with similar biomarkers (10 and 15), and cluster 12 expressing a few more biomarkers.

CD24, CLDN3, WFDC2, and TACSTD2 genes were present in all the tumour clusters of all samples, and the genes C1orf194, C20orf85, MS4A8, ODF3B, RSPH1, and TPPP3 appear to be co‐expressed.

The biomarkers found in tumour clusters were also tested in control samples, and the results are shown in Figure 1g,h. The expression of CD24 and TPPP3 in the controls is not surprising [25, 26], and the expression of other tumour biomarkers in control 3 could be due to a previous mechanical obstruction of the fallopian tube. However, this does not dispel all doubts that there could be pre‐tumour cells in control 3, supported by the presence of CLDN3 and WFDC2 which are two markers always present in tumour clusters.

### 3.2 Prediction of Endometrial Cancer Cells by Inference of Copy Number Variations

SCEVAN and CopyKAT software are the only tools capable of automatically distinguishing non‐malignant from malignant cells. The full profiles of the predicted CNVs of the tumour and control samples are shown in Figure S1 (SCEVAN) and Figure S2 (CopyKAT). In Figures S3 and S4 we report the predictions referring only to tumour clusters in each sample.

Variability in the number and location of inter‐ and intra‐patient CNVs was noted; however, CNV frequencies did not correlate with disease grade. Surprisingly, tumour cells were also identified in control samples with CNV frequencies comparable to those observed in patient samples.

**FIGURE 2:**
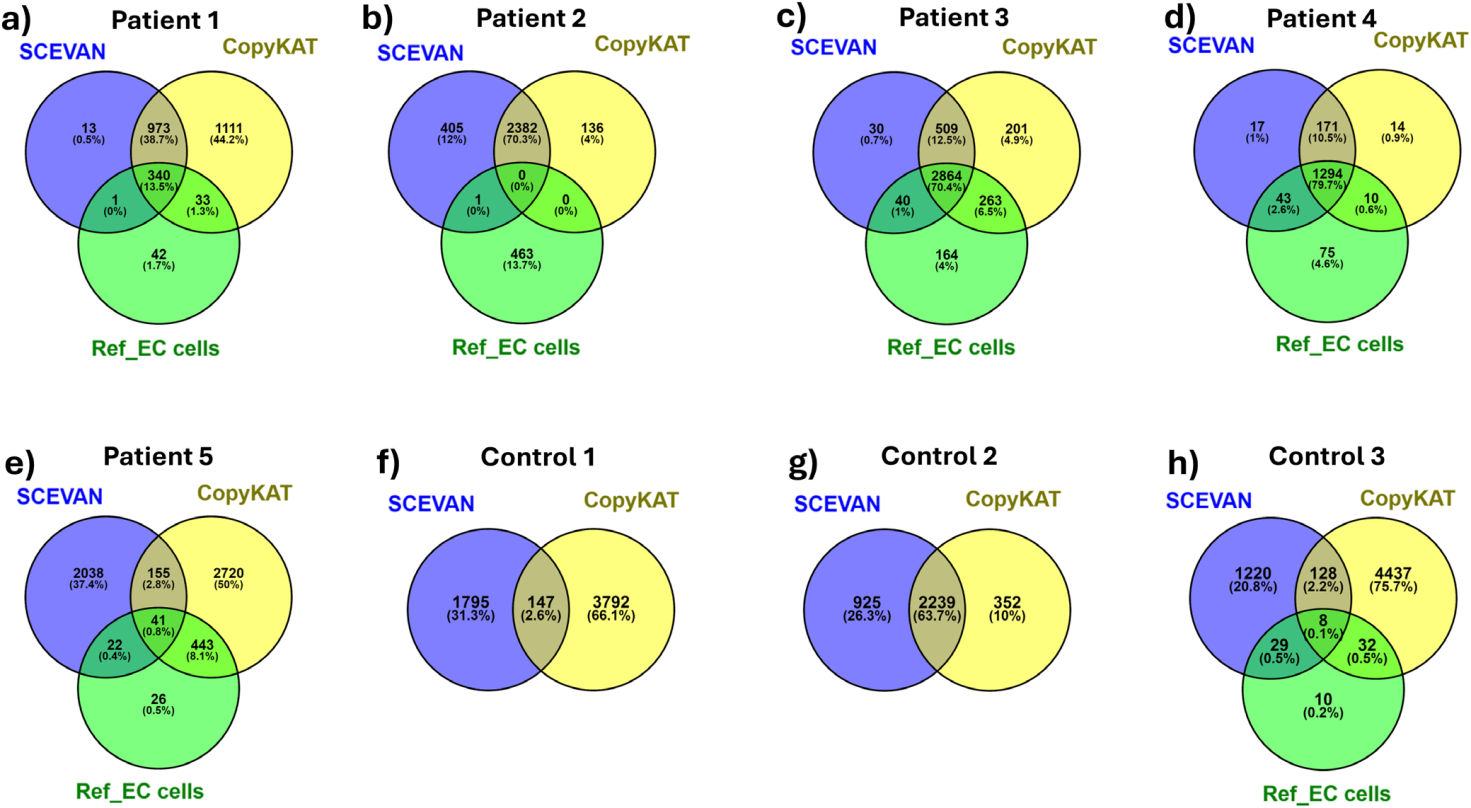
Venn diagram comparison between reference EC cells identified by biomarker expression and tumour cells according to SCEVAN and CopyKAT tools in each sample. In control samples 1 and 2, only the SCEVAN and CopyKAT tools predicted tumour cells. (a‐e) patients and (f‐h) controls.

We compared the predictions of CopyKAT and SCEVAN with the tumour cells identified by biomarkers using Venn diagrams (Figure 2).

Comparing results from the three prediction methods, it can be seen that most tumour cells identified in patients 1, 3, 4 and 5 by biomarkers have also been included in the SCEVAN and/or CopyKAT predictions. In patient 2 there was a good consistency between SCEVAN and CopyKAT but they seem to return only false positives, as nearly none of these cells were classified as tumoral based on biomarker analysis. In patient 5 there was a poor agreement between the tools as well as the cells considered tumorous. The predictions that showed greater consistency between the tools and cells considered cancerous were seen in patient 4. Unexpected results were observed in the controls (Figure 2f–h) in which 78% (5734/7310), 57% (3516/6143) and 83% (5854/7037) tumour cells were predicted in controls 1, 2 and 3 respectively. The ~64% agreement between the two tools for control 2 is particularly concerning because agreement between methods is often interpreted as increased confidence in the predictions.

The cell types identified by the SingleR tool in the tumour and control samples are shown in Table S4.

EC cells were mainly identified as epithelial, consistent with the hypothesis that EEC may originate from endometrial epithelial stem cells [27]. Table 1 reports predictions from the different methods of tumour cell identification with column 5 showing the number of tumour cells according to biomarkers, which are also epithelial according to SingleR. This represents a more general validation since EC cells are of epithelial origin; hence it is expected that most or all predicted tumour cells are also epithelial. Indeed, concordances greater than 88% and reaching up to 100% are noted, supporting the reliability of identification of tumour cells based on biomarkers. This comparison also supports the small proportion of tumour cells (~1%) in control 3 according to biomarkers. In general, large numbers of false positives were predicted by SCEVAN and CopyKAT, which seem to work better when a sample has a large number of tumour cells.

**TABLE 1:** CopyKAT and SCEVAN results.

Sample	Number of analysed cells in scRNA‐seq	Number of tumour cells according to biomarkers	Number of epithelial cells according to Single R	Number of tumour cells that are also epithelial	Number of tumour cells according to SCEVAN	Number of tumour cells according to CopyKAT
Patient 1	5697	416 (7.3%)	466 (8.2%)	413 (99.3%)	1327 (23.3%)	2457 (43.1%)
Patient 2	7963	464 (5.8%)	1248 (15.7%)	464 (100%)	2788 (35.0%)	2518 (31.6%)
Patient 3	6054	3331 (55%)	3817 (63.0%)	2952 (88.6%)	3443 (56.9%)	3837 (63.4%)
Patient 4	8110	1422 (17.5%)	1691 (20.9%)	1404 (98.7%)	1525 (18.8%)	1489 (18.4%)
Patient 5	8403	532 (6.3%)	785 (9.3%)	521 (97.9%)	2256 (26.8%)	3359 (40.0%)
Control 1	7310	0	37 (0.5%)	0	1942 (26.6%)	3939 (53.9%)
Control 2	6143	0	54 (0.9%)	0	3164 (51.5%)	2591 (42.2%)
Control 3	7037	79 (1.1%)	231 (3.3%)	59 (74.7%)	1385 (19.7%)	4605 (65.4%)

*Note:* The percentage in the column ‘Number of tumour cells that are also epithelial’ refers to the total number of tumour cells among epithelial cells (SingleR tool). Control 3 includes the number of cells found in the tumour cluster identified by biomarkers, as these cells exhibit a profile resembling that of cancer cells.

In Table 1 we see that the cells predicted by biomarkers (column 3) are almost entirely epithelial (column 5), respecting the necessary but not sufficient condition for the predicted cells to be tumorous. Unfortunately, SCEVAN and CopyKAT often predicted a number of tumour cells much higher than the number of epithelial cells, that is, the maximum number of potentially tumour cells. In other words, these tools returned an enormous number of false positives, and more specifically in some patients. Even more worrying is that false positives were also observed in large numbers in the control samples.

We further evaluated the sensitivity and specificity of SCEVAN and CopyKAT tools, considering the cells predicted tumoral by the marker‐based method as true positives (TP) and we report the calculations in Tables S5 and S6. Generally, CopyKAT showed the highest sensitivity and SCEVAN the highest specificity. Both tools showed inconsistency and reduced sensitivity in patient 2, while SCEVAN had low sensitivity in patient 5, resulting in an overall decline in its performance (Table S7).

### 3.3 Performance Analysis Referring to Individual Subclones

We also aimed to investigate the reliability of each predicted tumour subclone by SCEVAN and CopyKAT rather than the predicted single cells, to understand whether there are subclones entirely made up of tumour cells or not or whether a subclone can be made up of a variable number of tumour cells. In Table 2 we have reported the number of tumour and epithelial cells according to biomarkers and SingleR respectively, in each tumour subclone predicted by CopyKAT and SCEVAN tools.

**TABLE 2:** CopyKAT and SCEVAN performances.

	CopyKAT					SCEVAN				
	Sub clone	% Epithelial cells	% True tumour cells	SENS (referred to sample) (%)	SPEC (referred to sample) (%)	Sub clone	% Epithelial cells	% True tumour cells	SENS (referred to sample) (%)	SPEC (referred to sample) (%)
Pt1	1	99	96	83	100	1	99	93	47	100
						2	0	0	0	92
	2	2	1	7	61	3	0	0	0	96
						4	1	1	1	94
						5	100	99	35	100
Pt2	1	0	0	0	93	1	0	0	0	93
						2	2	0	0	83
	2	0	0	0	73	3	0	0	0	92
						4	1	0	0	90
Pt3	1	83	81	73	79	1	78	85	22	95
						2	99	82	23	94
	2	98	84	21	95	3	80	87	22	96
						4	97	83	21	95
Pt4	1	95	87	27	98	1	100	90	14	100
						2	97	90	30	99
	2	98	89	30	99	3	98	92	28	100
						4	90	79	19	99
						5	93	79	9	100
Pt5	1	65	53	90	95	1	2	1	2	89
						2	9	6	6	93
	2	1	0	1	74	3	5	3	2	95
						4	22	3	2	95
Ctrl1	1	0	0	—	79	1	0	0	—	94
						2	0	0	—	97
	2	0	0	—	67	3	0	0	—	94
						4	0	0	—	98
						5	0	0	—	91
Ctrl2	1	0	0	—	77	1	0	0	—	76
	2	0	0	—	81	2	0	0	—	85
						3	0	0	—	88
Ctrl3	1	0	0	4	71	1	3	3	14	95
						2	30	6	25	96
	2	4	2	47	69	3	0	0	1	94
						4	0	2	6	97

*Note:* Comparative table of sensitivity (SENS) and specificity (SPEC) of the tumour and control samples between CopyKAT and SCEVAN tools, together with the percentage of tumour and epithelial cells in each tumour subclone. The sensitivity and specificity of a certain subclone were calculated assuming that a tool indicated only that particular subclone as tumorous.

Given that a necessary but not sufficient condition for the cells indicated as cancerous to be acceptable is that they are epithelial, most of the subclones were either almost completely made up of tumour cells or not, and the percentage of these cells was similar or slightly lower than that of epithelial cells. In patient 1, only CopyKAT subclone 1 was correctly identified as a tumour subclone, and it alone captured almost all of the patient's tumour cells. It was completely made up of epithelial cells and this could have been an indication of the reliability of the prediction. Subclone 2 was almost devoid of tumour cells as well as epithelial cells, indicating the poor reliability of this clone. For SCEVAN, subclones 1 and 5 seemed to be correctly predicted as tumorous, as proven by their strong epithelial composition. Subclones 2, 3 and 4 were clearly false positives as supported by the absence of epithelial cells. The subclones predicted in patient 2 are of concern as they constitute false positives, but fortunately, the absence of epithelial cells might have been an indication for the user to discard these predictions. The subclones in patients 3 and 4 all had a high content of tumour cells as well as epithelial cells. Only one subclone in patient 5, predicted by CopyKAT, was reliable, whereas none of the subclones predicted by SCEVAN were reliable. All subclones in controls 1 and 2 were false positives, as could be deduced from the absence of epithelial cells. In control 3, reliable subclones showed poor sensitivity.

### 3.4 Prediction of Endometrial Cancer Cells by Copy Number Variation Score

InferCNV and sciCNV tools do not directly predict malignant cells based on inferred CNVs; therefore the user must try to identify cancer cells by calculating the CNV score of each cell for each CNV region detected. Figure S5 shows the heatmaps of each sample (tumour and control) obtained by the InferCNV tool, whereas Figure S6 shows beanplots of the CNV scores of each sample calculated by sciCNV. Approximately 10%, 75% and 30% of cells from patients 1, 3 and 4 respectively, presented amplifications in correspondence with the long arm of chromosome 1. Similar percentages of cells, with the same rearrangements, were highlighted by SCEVAN (Figure S1) and CopyKAT (Figure S2) with both tools predicting them as tumorous.

**FIGURE 3:**
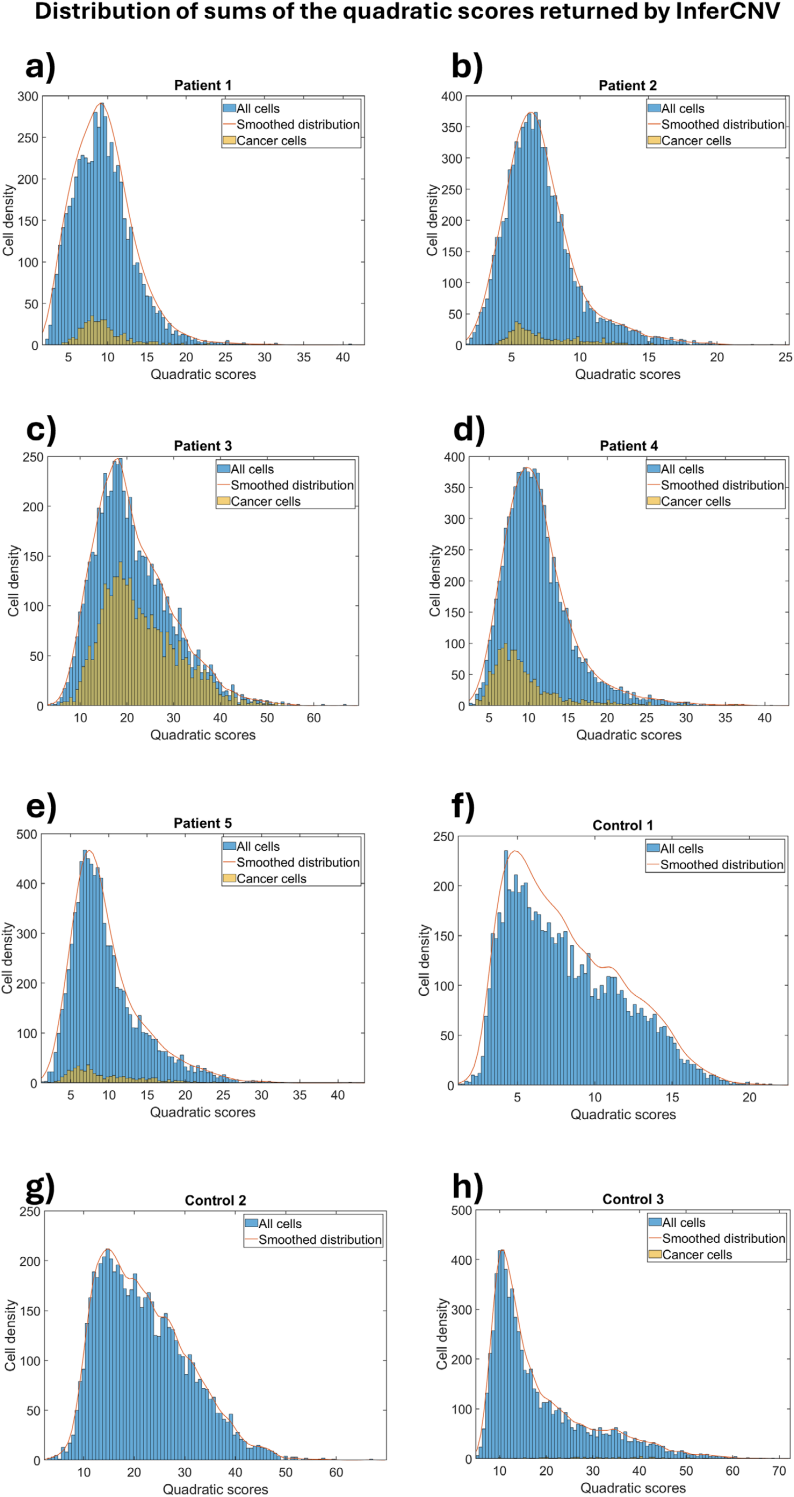
Distribution of the sums quadratic scores obtained by InferCNV's CNV matrix and centred at 0. Non‐malignant cells are represented by blue columns, while EC cells identified by biomarkers are depicted by yellow columns. (a–e) plot of cell density versus quadratic sum of patient 1–5. (f–h) plot of cell density versus quadratic sum of control 1–3.

From InferCNV's CNV_matrix, we calculated the quadratic sum of the scores of the predicted CNVs for each gene, obtaining a total CNV score for each cell. Figure 3 shows distributions of the total CNV scores of all cells (cancerous and non‐cancerous) in each sample; the total CNV scores of tumour cells identified by biomarkers are shown in yellow. In this way it is possible to try to identify a threshold, in terms of total CNV score, beyond which to consider a cell as tumorous. One would expect that tumour cells should be separated from normal cells and located to the right of the graph because they are thought to have a greater number of CNVs than normal cells.

However, the results we obtained differed from those expected, as a bimodal distribution was not observed. Furthermore, the tumour cell populations completely overlapped with the other cells; that is, they have similar total CNV scores.

**FIGURE 4:**
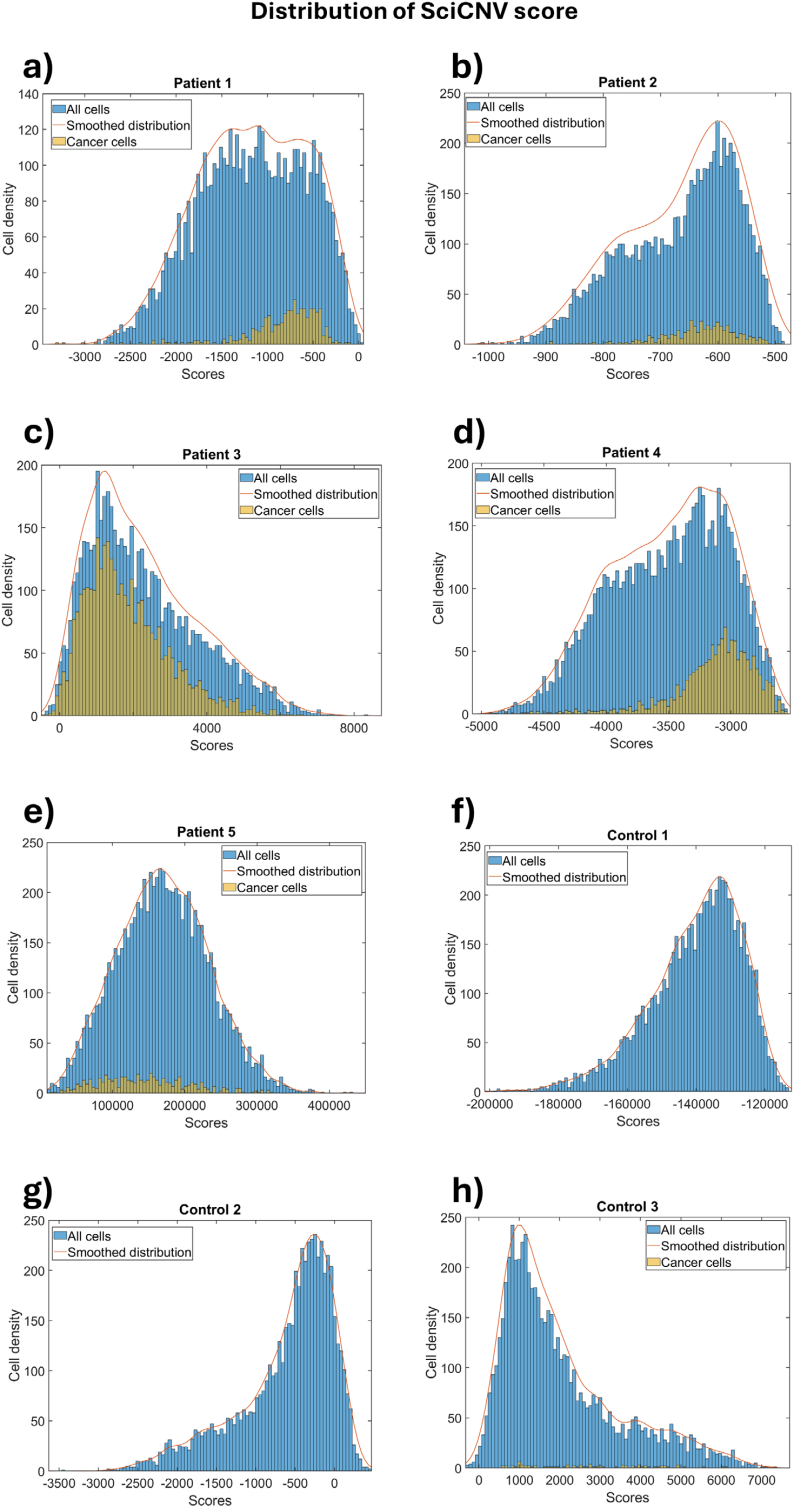
Distribution of CNV scores obtained by sciCNV tool. Non‐malignant cells are represented by blue columns, while EC cells identified by biomarkers are depicted by yellow columns. (a–e) plot of cell density versus quadratic sum of patient 1–5. (f–h) plot of cell density versus quadratic sum of control 1–3.

SciCNV returns a total CNV score without the need for calculation by the user. However, it uses a different formula than that of InferCNV, and therefore the two scores are not directly comparable. Unfortunately, the tumour cells do not appear separated from the rest, so it was not possible to identify a discriminating threshold (Figure 4).

## 4 Discussion

This study arises from the observation that in the Materials and Methods sections of studies dealing with the analysis of scRNA‐seq data, it is written that the identification of tumour cells in a sample is accomplished through the use of biomarkers and validation using tools that infer the presence of CNVs. The use of CNVs is based on the fact that ‘CNVs are a hallmark of all cancers’. There are at least four tools for inferring CNVs starting from scRNA‐seq expression data but there are no indications of which is the best tool. Moreover, how to perform validation using CNVs predictions is not specified; therefore, it is not clear whether validation means considering the cells predicted by both methods (biomarkers and presence of CNVs) as tumorous.

In this study different methods of identifying EC cells from single‐cell sequencing experiments were compared. The identification of tumour cells used as references was done by EC biomarker genes that we collected from the literature. This method may not have identified all EC cancer cells or included some non‐cancerous cells, but this is unavoidable because on one hand, the shallow sequencing of scRNA‐seq may not have captured all the genes actually expressed, and on the other hand it is not known how many and which biomarkers are necessary and sufficient to define a cancer cell. It was unexpected to find CD24 and TPPP3 biomarkers expressed in control samples. However, this could have been caused by the secretory phase of the uterine cycle at the time of sampling, specifically during the implantation window (WOI) [25, 26]. In this sense we highlight the need for single‐cell RNA‐seq data from patients with cells ascertained to be tumorous so that they can be used as a reference in future studies.

We carried out comparisons among reference EC cells identified by biomarkers and those obtained using the CNVs inference tools, SCEVAN, CopyKAT, InferCNV and sciCNV. We observed that SCEVAN and CopyKAT overestimate the number of tumour cells more than half the time, even up to six times more. Unfortunately, this overestimation does not always correspond to a high sensitivity; for example, although more than 2500 tumour cells were predicted in a patient, none were actually cancerous. Furthermore, in the control samples in which no tumour cells are expected, up to approximately 4000 tumour cells were predicted.

InferCNV and sciCNV predict CNVs but do not indicate which cells are tumorous, and even though we calculate the CNV scores or use those provided by sciCNV, we demonstrate that it is not possible to identify a CNV score threshold beyond which to consider a cell as tumorous. Furthermore, the distributions of the CNV scores predicted by InferCNV and sciCNV for each cell show that the tumour cells identified by biomarkers are not distributed towards the right side of the graph, where higher scores indicate a greater presence of CNVs. Since the distribution of tumour cells is indistinguishable from that of normal cells, many cells are tumorous despite having a low number of predicted CNVs and this is in agreement with other experimental data that show a low number of CNVs in EC compared to other tumours [28].

Cells with few CNVs may be cancerous due to the accumulation of mutations rather than an increase in the number of CNVs. Alternatively, tumorigenesis could be due to the presence of few CNVs which, involve cancer driver genes. This raises the risk that tumour cells with low CNV scores might be discarded at the validation stage.

CopyKat and SCEVAN tools, which directly predict tumour cells, produce a large number of false positives as indicated by the higher number of predicted tumour cells compared to epithelial cells. Therefore, we suggest considering the cells predicted as tumorous to also be epithelial.

It should be noted that the number of samples used in this study may be a limitation, but we must also note that our conclusions are found in all the samples that were analysed. We emphasise the lack of agreement among the tools and their limited correspondence with the expected results. We suggest caution with the automated utilisation of these tools, and until more accurate algorithms become available, we recommend filtering predictions to ensure that the predicted EC cells are at least classified as epithelial, a necessary but not sufficient condition.

## Author Contributions

**Erica Dugo:** data curation (equal), formal analysis (equal), investigation (equal), writing – original draft (equal). **Francesco Piva:** conceptualization (equal), supervision (equal), writing – review and editing (equal). **Matteo Giulietti:** data curation (equal), methodology (equal), writing – review and editing (equal). **Luca Giannella:** data curation (equal), methodology (equal). **Andrea Ciavattini:** conceptualization (equal), supervision (equal), writing – review and editing (equal).

## Ethics Statement

The authors have nothing to report.

## Consent

The authors have nothing to report.

## Conflicts of Interest

The authors declare no conflicts of interest.

## Supporting information


**Table S1:** Characteristics of analysed specimens.
**Table S2:** Overexpressed genes in Endometrial Cancer.
**Table S3:** Comparison of CNV inference tools from scRNA‐seq.
**Table S4:** Cell type annotation by SingleR software.
**Table S5:** SCEVAN performance.
**Table S6:** CopyKAT performance.
**Table S7:** Sensitivity and specificity of the SCEVAN and CopyKAT tools.
**Figure S1:** Heatmaps of CNVs predicted by SCEVAN tool.
**Figure S2:** Heatmaps of CNVs predicted by CopyKAT tool.
**Figure S3:** Heatmaps of CNVs predicted in tumour cells by SCEVAN tool.
**Figure S4:** Heatmaps of CNVs predicted in tumour cells by CopyKAT tool.
**Figure S5:** Heatmaps CNVs predicted by InferCNV tool in each sample.
**Figure S6:** Beanplot of the CNV scores obtained by sciCNV tool.

## Data Availability

Public data used in this work can be obtained from the Gene Expression Omnibus (GEO) database at https://www.ncbi.nlm.nih.gov/geo/, reference numbers GSE173682 and GSE183837.
