# Supplementary material for: Tool Comparison for Detecting Tumour Cells in Endometrial Cancer via Single‐Cell Copy Number Variations Analysis
Source: J Cell Mol Med. 2025 Oct 29;29(21):e70932. doi: 10.1111/jcmm.70932 (PMC12571190; doi:10.1111/jcmm.70932)

Tool comparison for detecting tumour cells in endometrial cancer via single-cell copy number variations analysis

Supporting Information

Supplementary methods

Tools to predict copy number variations

Single CEll Variational Aneuploidy aNalysis (SCEVAN)

SCEVAN tool (version 1.0.1) infers CNVs and uses them to automatically detect malignant and non-malignant cells through a joint segmentation algorithm. This algorithm works simultaneously on all cells to identify breakpoints shared among those belonging to the same clone. Segmentation is optimised using the Mumford–Shah energy model, an original computer vision model adapted to genomic data analysis.

Copy number Karyotyping of Aneuploid Tumors (CopyKAT)

CopyKAT tool (version 1.1.0) has the ability to automatically distinguish aneuploid malignant cells from diploid non-malignant cells by inferring CNVs with an average resolution of 5 Mb. In particular, to obtain stable and reliable segmentations, CopyKAT applies a combination of statistical models, including the Freeman–Tukey Transformation to stabilise variance, a dynamic linear model to smooth data, and a Poisson-Gamma model with Bayesian inference via Markov Chain Monte Carlo (MCMC) to estimate copy number profiles. The segmentation is then refined through a Kolmogorov–Smirnov test (controlled by the KS.cut parameter), which allows adjacent gene windows that do not show significant differences to be merged.

Inferring Copy Number Variations (InferCNV)

InferCNV tool (version 1.16.0) employs a comparative approach by analysing gene expression intensity relative to the chromosomal position of reference "normal" cells. CNV inference is performed by comparing the expression intensity of genes of the samples to be tested versus those of 'normal' cells. The raw expression matrix is first levelled using a sliding window (default setting: 101 genes) to reduce noise and highlight regional expression changes indicative of CNVs. A hidden Markov model (HMM) is then applied to assign discrete CNV states (deletion, neutral, amplification) along the genome. To increase robustness, a Bayesian maximum likelihood filter is used to retain only high-confidence CNV calls.

Inferring single-cell CNV (sciCNV)

sciCNV tool (version 0.99.73) requires the input of a subset of normal cells to be used as the reference for inferring CNVs. The input to sciCNV is a raw expression matrix normalised using the RTAM1 and RTAM2 methods, which were developed specifically to improve gene expression alignment between single cells. These methods are based on the idea that highly expressed genes are detected more accurately and allow for a more accurate estimation of expression differences due to copy number variations. RTAM ranks genes based on their expression in each cell and uses cell-specific non-linear adjustments to standardise expression intensities, thereby minimising the stochastic noise and dropout typical of scRNA-seq data. After

normalisation, sciCNV calculates a CNV score for each cell, based on the moving average of expression discrepancies relative to the reference and the integration of estimates obtained from RTAM1 and RTAM2. These steps allow for the sensitive identification of copy number variations even at resolutions between 19 and 46 Mb.

Supplementary table 1 (Table S1):
Characteristics of analysed specimen. AA (African American), CAU (Caucasian) and AC (Asian).

Sample	*Histology and Stage*	*Grade*	*Age*	*Race*	*GEO id*
Patient 1	EEC IA	2	70	AA	GSM5276933
Patient 2	EEC IA	3	70	CAU	GSM5276934
Patient 3	EEC IA	2	70	CAU	GSM5276935
Patient 4	EEC IA	1	49	CAU	GSM5276936
Patient 5	EEC IA	1	62	CAU	GSM5276937
Control 1					GSM5572238
Control 2	Not tumour		29-35	AC	GSM5572239
Control 3					GSM5572240

Supplementary table 2 (Table S2):
Overexpressed genes in Endometrial Cancer. Immunohistochemistry (IHC), Single cell RNA-sequencing (scRNA-seq), Electrochemiluminescence (ECL), Enzyme Immunoassay (EIA) and Immunofluorescence (IF). The EC tumour biomarkers provided in Table S2 do not provide a precise identikit of tumour cells since the markers which are necessary, their potential interchangeability, and the minimum number required are not known. Additionally, there is no established formula that weighs the presence or absence of each marker in each cell to determine a final score or threshold for identifying cancerous cells.

Endometrial Cancer	*Overexpressed Gene Markers*	*Sample Information*	*Techniques*	*Reference*	*PMID*
Endometrioid Endometrial Cancer	CLDN3	30 tissue samples	IHC and Real-time PCR	Pan et al. 2007	17291259
Endometrial Cancer	MMP7	197 tissue samples	IHC	Misugi et al. 2005	16142384
Endometrial Cancer	LCN2	316 tissue samples	IHC	Mannelqvist et al. 2012	22559235
Serous carcinoma, Clear Cell carcinoma and Endometrioid Endometrial Cancer	LCN2	85 tissue samples	IHC	Mihalj et al. 2014	25522256
Endometrioid Endometrial Cancer	LCN2 and SAA1/2	5 tissue samples	scRNA-seq	Ren et al. 2022	36273006
Endometrioid Endometrial Cancer, Serous carcinoma, Mixed carcinoma (endometrioid and clear cell), Clear Cell carcinoma, and Uterine Carcinosarcomas	MUC1	310 tissue samples	IHC	Morrison et al. 2007	18091386
Endometrial Cancer	MUC16 and WFDC2	150 tissue samples	ECL	Dong et al. 2017	29067084
Endometrioid Endometrial Cancer and Serous carcinoma	WFDC2	Ark 2 and HEC-1B cell line and tumour Tissues	IHC and Real-time PCR	Li et al. 2013	23502467
Endometrial Cancer	WFDC2	143 blood specimens	EIA	Blackman et al. 2021	34957977
Endometrioid Endometrial Cancer	MUC16, WFDC2, IMPA2, CD24 and SOX9	5 tissue samples	scRNA-seq	Regner et.al 2021	34739872
Endometrial Cancer	SOX9	Ishikawa and Hec 251 cell line and 55 tissues samples	IHC	Saegusa et al. 2012	22698986

Endometrial Cancer	RSPH1	5 tissue samples	scRNA-seq	Guo et al. 2021	33429363
Endometrioid Endometrial Cancer	TACSTD2	131 tissue samples	IHC	Bignotti et al. 2011	21892093
Endometrial Cancer	TPPP3	Ishikawa, KLE, RL-95-2 and AN3CA cell line and 15 blood and tissue samples	RT-PCR and IF	Shen et al. 2021	33644928
Endometrial Cancer	ADAMTS19, ADAMTS8, AGTR2, AKAP14, ALPG, ALPP, AMBN, ANKRD66, AOC1, AP1S2, APOBEC4, AQP5, ARMC3, ASB12, ASIC2, ASRGL1, BCAT1, BEX5, C11orf16, C11orf97, C1orf194, C1orf87, C20orf85, C22orf15, C2orf88, C4orf54, C5orf49, C6orf118, C9orf24, CA8, CACNG6, CALCB, CAPN13, CAPN6, CAPS, CAPSL, CATSPERD, CCDC146, CCDC17, CCDC33, CCDC78, CCNA1, CDC20B, CDHR4, CEACAM21, CFAP100, CFAP107, CFAP141, CFAP157, CFAP161, CFAP276, CFAP43, CFAP45, CFAP47, CFAP52, CFAP57, CFAP65, CFAP73, CFAP74, CFAP77, CIBAR2, CLPSL1, CLXN, CNGA4, COL26A1, COL9A1, CREB3L4, CROCC2, CRYBA4, CRYBB2, CRYGC, CST4, CTNNA2, CTXN1, DACT2, DIRAS3, DKK4, DLX5, DLX6, DNAAF1, DNAAF3, DNAAF6, DNAH12, DNAH9, DNAI1, DNAI2, DNAI3, DRC1, DRC7, DYDC1, DYDC2, DYNLT4, ECEL1, EMID1, EMX2, ENKUR, ENSG00000230707, ERAS, ERBB4, ERICH3, ESR1, EYA2, FAM166B, FAM181A, FAM183A, FAM216B, FAM81B, FBLN1, FGF18, FGF20, FGF3, FGF8, FGF9, FOXB1, FOXJ1, FOXN4, FZD10, GABRP, GAD1, GALP, GAS2L2, GATA2, GDF5, GFRA3, GFRA4, GLYATL2, GLYATL3, GRIA2, GSTA3, HBA1, HOATZ, HOXB5, HOXB6, HOXB8, HOXB9, IGHD2-15, IGHD3-22, IGHD7-27, IGLJ2, IGLJ3, IGLJ6, IGLJ7, IGLL1, IHH, IL19, JSRP1, KCNH3, KCNRG, KLHL14, KRT27, KRT28, KRT71, KRTAP11-1, LDLRAD1, LEFTY1, LGR5, LHFPL7, LKAAEAR1, LRCOL1, LRP4, LRRC10B, LRRC18, LRRC3B, LRRC46, LRRC71, LRRN2, LRRTM1, LTF, MAP2K6, MAP3K19, MCCD1, MEGF11, MMP26, MORN5, MS4A8, MSX1, MSX2, MT4, MYH7B, NAT8L, NEUROG2, NLGN4X, NPAS3, OBP2B, ODAD1, ODF3B, OMG, OR1N1, OVGP1, PACSIN1, PAX2, PAX8, PCDH19, PGR, PIERCE1, PKHD1L1, PMCH, PRLH, PRPH, PRR9, PRSS56, PYY, PZP, RHEX, RIIAD1, RNF183, ROPN1L, RSPH1, RSPH4A, RXFP1, SCGB1D1, SCGB1D2, SCGB1D4, SCGB2A1, SCGB3A1, SCX, SIAH3, SLC10A4, SLC22A16, SLC23A1, SLC25A35, SMIM6, SNTN, SOHLH1, SOX17, SP9, SPEF1, SPINK9, SRARP, SRD5A2, STMND1, STOML3, STX18, TCTE1, TEKT1, TEKT2, TEKT4, TGM7, TMEM178A, TMEM190, TMEM26, TPH1, TPPP3, TRH, TSNAXIP1, TTC29, TTLL10, TUBA4B, UCMA, VAX2, VTCN1, VWA3A, VWA3B, VWA5B2, WDR38, WFDC2, WFDC6, WIF1, WNT7A, ZBBX and ZMYND10				The Human Protein Atlas

Supplementary table 3 (Table S3):

Comparison of CNV inference tools from scRNA-seq. Summary of the main computational tools used for inferring CNVs from scRNA-seq data, highlighting their core approaches, modeling strategies, normalization procedures, and key strengths.

Method	Main Approach	Segmentation /Modeling	Normalization and Pre-processing	Key Strengths
SCEVAN (version 1.0.1)	Joint variational segmentation applied simultaneously to all cells	Mumford–Shah energy model (variational)	Standard, works directly on expression data	Precise segmentation with shared breakpoints, robust clonal analysis
CopyKAT (version 1.1.0)	Probabilistic approach with hierarchical clustering	Poisson-Gamma model + MCMC + Kolmogorov–Smirnov test	Freeman-Tukey transformation and polynomial smoothing	High resolution (~5 Mb), strong statistical robustness
InferCNV (version 1.16.0)	Comparison of gene expression to reference "normal" cells	Moving average + Hidden Markov Model (HMM)	No specific normalization; uses biological reference cells	Effective use of real reference cells, useful for tumor subpopulation

				detection
sciCNV (version 0.99.73)	Comparison to reference “normal” cells with RTAM1/RTAM2 normalization	Moving average on normalized expression + CNV score calculation	RTAM1 and RTAM2 normalization, tailored for scRNA-seq data	Optimized normalization, high sensitivity to subtle expression changes

Supplementary table 4 (Table S4):

Cell type annotation by SingleR software. Endothelial, epithelial and immune cells are in higher percentages in diseased tissues than in healthy ones. On the contrary, fibroblasts are present in greater quantities in samples from healthy subjects. However, large variations in cell composition may be due to the different phases of the uterine cycle. The “other cells” consist of those foreign to the endometrium (e.g., astrocyte, bone marrow, gametocytes, hepatocytes, keratinocytes, hematopoietic stem cells) probably due to a not high specificity of SingleR.

	**Cell type and cell count (%)**							
	**Patient 1**	**Patient 2**	**Patient 3**	**Patient 4**	**Patient 5**	**Control 1**	**Control 2**	**Control 3**
IMMUNE CELLS	704 (12.35%)	1378 (17.31%)	849 (14.02%)	2931 (36.14%)	1797 (21.39%)	350 (4.79%)	686 (11.17%)	749 (10.64%)
NON IMMUNE CELLS	4992 (87.62%)	6585 (82.69%)	5205 (85.98%)	5179 (63.86%)	6606 (78.61%)	6960 (95.21%)	5457 (88.83%)	6288 (89.36%)
Astrocyte	1 (0.02%)	0	5 (0.08%)	0	2 (0.02%)	2 (0.03%)	3 (0.05%)	1 (0.01%)
Bone Marrow Cells	0	0	1 (0.02%)	0	3 (0.04%)	1 (0.01%)	0	0
Chondrocytes	117 (2.05%)	26 (0.33%)	2 (0.03%)	12 (0.15%)	71 (0.84%)	15 (0.21%)	78 (1.27%)	38 (0.54%)
Embryonic Stem Cells	1 (0.02%)	3 (0.04%)	27 (0.45%)	7 (0.09%)	0	3 (0.04%)	4 (0.07%)	7 (0.10%)
Endothelial Cells	1599 (28.07%)	1136 (14.27%)	106 (1.75%)	258 (3.18%)	835 (9.94%)	42 (0.57%)	41 (0.67%)	186 (2.64%)
Epithelial Cells	466 (8.18%)	1248 (15.67%)	3817 (63.05%)	1691 (20.85%)	785 (9.34%)	37 (0.51%)	54 (0.88%)	231 (3.28%)
Erythroblast	0	0	0	0	2 (0.02%)	2 (0.03%)	1 (0.02%)	0

Fibroblasts	591 (10.37%)	809 (10.16%)	208 (3.44%)	973 (12.00%)	1797 (21.39%)	3878 (53.05%)	2984 (48.58%)	3429 (48.73%)
Gametocytes	0	0	3 (0.05%)	1 (0.01%)	1 (0.01%)	0	0	0
Hepatocytes	0	2 (0.03%)	1 (0.02%)	0	1 (0.01%)	0	0	1 (0.01%)
Hemapoietic Stem Cells CD34+	3 (0.05%)	7 (0.09%)	1 (0.02%)	1 (0.01%)	6 (0.07%)	2 (0.03%)	0	3 (0.04%)
Hemapoietic Stem Cells-G-CSF	1 (0.02%)	1 (0.01%)	2 (0.03%)	0	0	0	0	1 (0.01%)
Induced Pluripotent Stem Cells	1 (0.02%)	1 (0.01%)	343 (5.67%)	13 (0.16%)	1 (0.01%)	6 (0.08%)	6 (0.10%)	3 (0.04%)
Keratinocytes	1 (0.02%)	0	0	0	1 (0.01%)	0	0	2 (0.03%)
Megakaryocyte-Erythroid Progenitors	0	0	0	0	1 (0.01%)	0	0	0
Mesenchymal Stem Cells	59 (1.04%)	33 (0.41%)	7 (0.12%)	8 (0.10%)	36 (0.43%)	78 (1.07%)	192 (3.13%)	12 (0.17%)
Myelocyte	0	0	0	0	1 (0.01%)	0	0	0
Neuroepithelial Cells	0	0	22 (0.36%)	0	2 (0.02%)	1 (0.01%)	1 (0.02%)	3 (0.04%)
Neurons	7 (0.12%)	17 (0.21%)	39 (0.64%)	8 (0.10%)	33 (0.39%)	27 (0.37%)	72 (1.17%)	14 (0.20%)
Osteoblasts	9 (0.16%)	9 (0.11%)	1 (0.02%)	9 (0.11%)	11 (0.13%)	6 (0.08%)	2 (0.03%)	14 (0.20%)
Platelets	0	0	0	0	1 (0.01%)	0	0	0
Pro-Myelocyte	0	0	0	0	1 (0.01%)	2 (0.03%)	0	1 (0.01%)
Smooth Muscle Cells	550 (9.65%)	1200 (15.07%)	123 (2.03%)	453 (5.59%)	990 (11.78%)	1303 (17.82%)	1350 (21.98%)	837 (11.89%)
Tissue Stem Cells	1586 (27.84%)	2093 (26.28%)	497 (8.21%)	1745 (21.52%)	2025 (24.10%)	1555 (21.27%)	669 (10.89%)	1505 (21.39%)

Supplementary table 5 (Table S5):

SCEVAN performance. The table shows the true positives (TP), false positives (FP), true negatives (TN) and false negatives (FN) values for calculating the sensitivity and specificity of SCEVAN. The column labelled "Biomarkers" shows the tumour cell count for each sample detected by the marker method.

Sample	Biomarkers	SCEVAN						
		TP	FP	TN	FN	Sensitivity	Specificity	Total cells
Patient 1	416	341	986	4295	75	0.82	0.81	5697
Patient 2	464	1	2787	4712	463	0.00	0.63	7963
Patient 3	3331	2904	539	2184	427	0.87	0.80	6054
Patient 4	1422	1337	188	6500	85	0.94	0.97	8110
Patient 5	532	63	2193	5678	469	0.12	0.72	8403
Control 1	0	0	1942	5368	0	-	0.73	7310
Control 2	0	0	3164	2979	0	-	0.48	6143
Control 3	79	37	1348	5610	52	0.47	0.81	7037
					Mean value	0.54	0.75	

Supplementary table 6 (Table S6):

CopyKAT performance. The table shows the true positives (TP), false positives (FP), true negatives (TN) and false negatives (FN) values for calculating the sensitivity and specificity of CopyKAT. The column labelled "Biomarkers" shows the tumour cell count for each sample detected by the marker method.

Sample	Biomarkers	CopyKAT						
		TP	FP	TN	FN	Sensitivity	Specificity	Total cells
Patient 1	416	373	2084	3197	43	0.90	0.61	5697
Patient 2	464	0	2518	4981	464	0.00	0.66	7963
Patient 3	3331	3127	710	2013	204	0.94	0.74	6054
Patient 4	1422	1304	185	6503	118	0.92	0.97	8110
Patient 5	532	484	2875	4996	48	0.91	0.63	8403
Control 1	0	0	3939	3371	0	-	0.46	7310
Control 2	0	0	2591	3552	0	-	0.58	6143
Control 3	79	40	4565	2392	39	0.51	0.34	7037
					Mean value	0.70	0.62	

Supplementary table 7 (Table S7):
Sensitivity and specificity of the SCEVAN and CopyKAT tools.

Sample	Biomarkers	SCEVAN		CopyKAT	
		Sensitivity	Specificity	Sensitivity	Specificity
Patient 1	416	0.82	0.81	0.9	0.61
Patient 2	464	0	0.63	0	0.66
Patient 3	3331	0.87	0.8	0.94	0.74
Patient 4	1422	0.94	0.97	0.92	0.97
Patient 5	532	0.12	0.72	0.91	0.63
Control 1	0	-	0.73	-	0.46
Control 2	0	-	0.48	-	0.58
Control 3	79	0.47	0.81	0.51	0.34
	Mean value	0.54	0.74	0.7	0.62

Supplementary figure 1 (Figure S1):
Heatmaps of CNVs predicted by SCEVAN tool. Note that all cells were reported, and they are clustered in two groups: cancerous (red in the ordinate axis) and healthy (green in the ordinate axis). In addition, the heatmap generated by SCEVAN shows the inferred CNVs in 22 chromosomes of each sample: orange indicates amplifications, blue deletions and white neutral states.

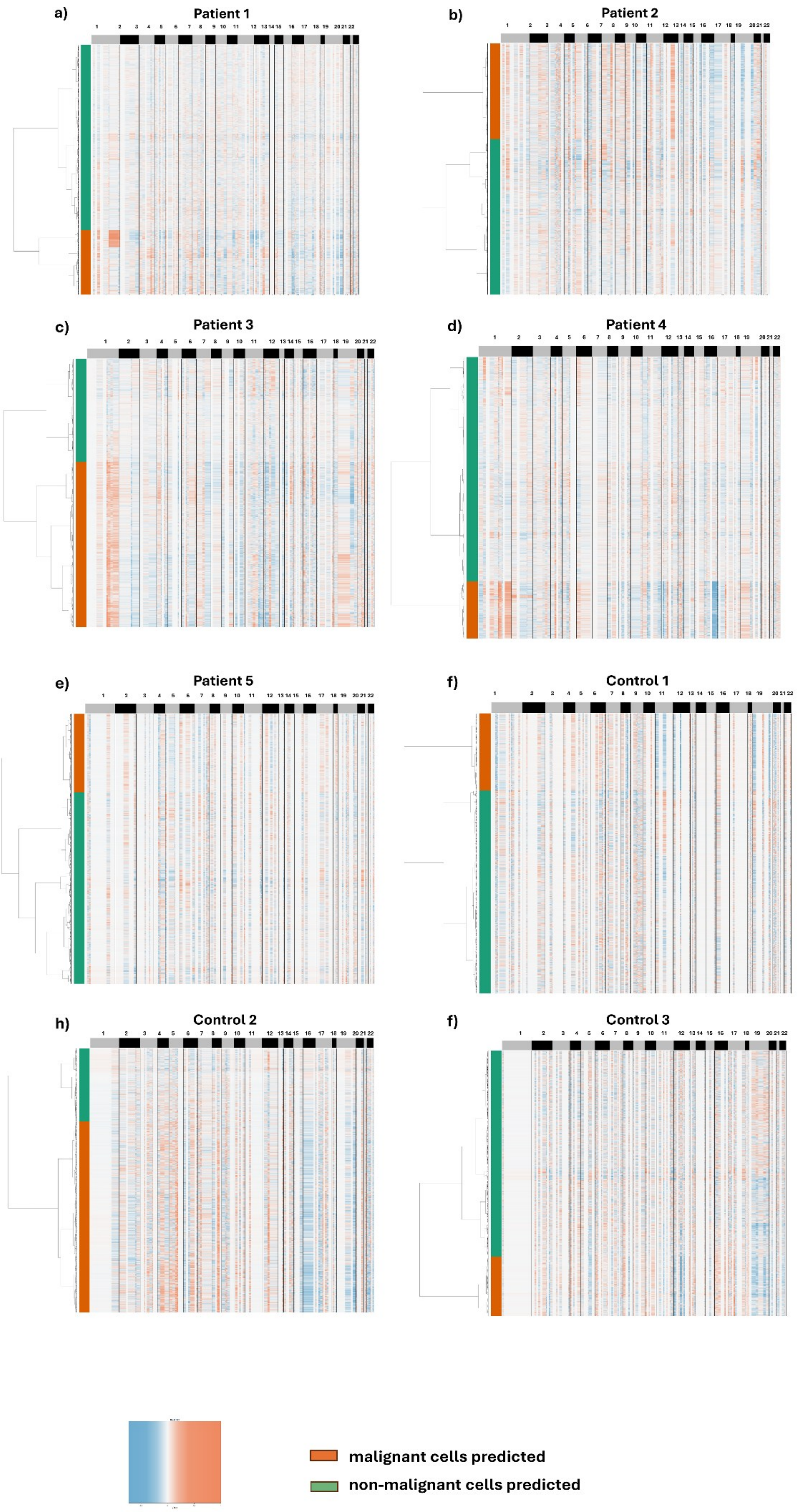
a)
Patient 1
b)
Patient 2
c)
Patient 3
d)
Patient 4
e)
Patient 5
f)
Control 1
h)
Control 2
f)
Control 3
malignant cells predicted
non-malignant cells predicted

Supplementary figure 2 (Figure S2):

Heatmaps of CNVs predicted by CopyKAT tool. Note that all cells were reported, and they are clustered in two groups: cancerous (red in the ordinate axis) and healthy (green in the ordinate axis). In addition, the heatmap generated by CopyKAT shows the inferred CNVs in 22 + X chromosomes of each sample: orange indicates amplifications, blue deletions and white neutral states. In patient 1, each software identified amplifications in a large region mapping on the long arm of chromosome 1 and distributed deletions in a few chromosomes. However, only SCEVAN detected deletions in chromosomes 19. In patient 2, more marked amplifications were detected in chromosome 12 by both methods. In patient 3 there were amplifications in chromosomes 1 and 19 according to SCEVAN, along with deletions in chromosome 19. Interestingly, CopyKAT revealed a more distinct copy number variation pattern in the predicted diploid cells compared to the aneuploid cells. In patient 4, SCEVAN and CopyKAT detected amplifications in a large region of chromosome 1. However, SCEVAN also detected more deletions in chromosomes 16, while CopyKAT detected deletions in chromosomes 11 and 17. The results generated by SCEVAN and CopyKAT revealed that patient 5 had a uniform CNV profile in all chromosomes, with no significant amplifications or deletions.

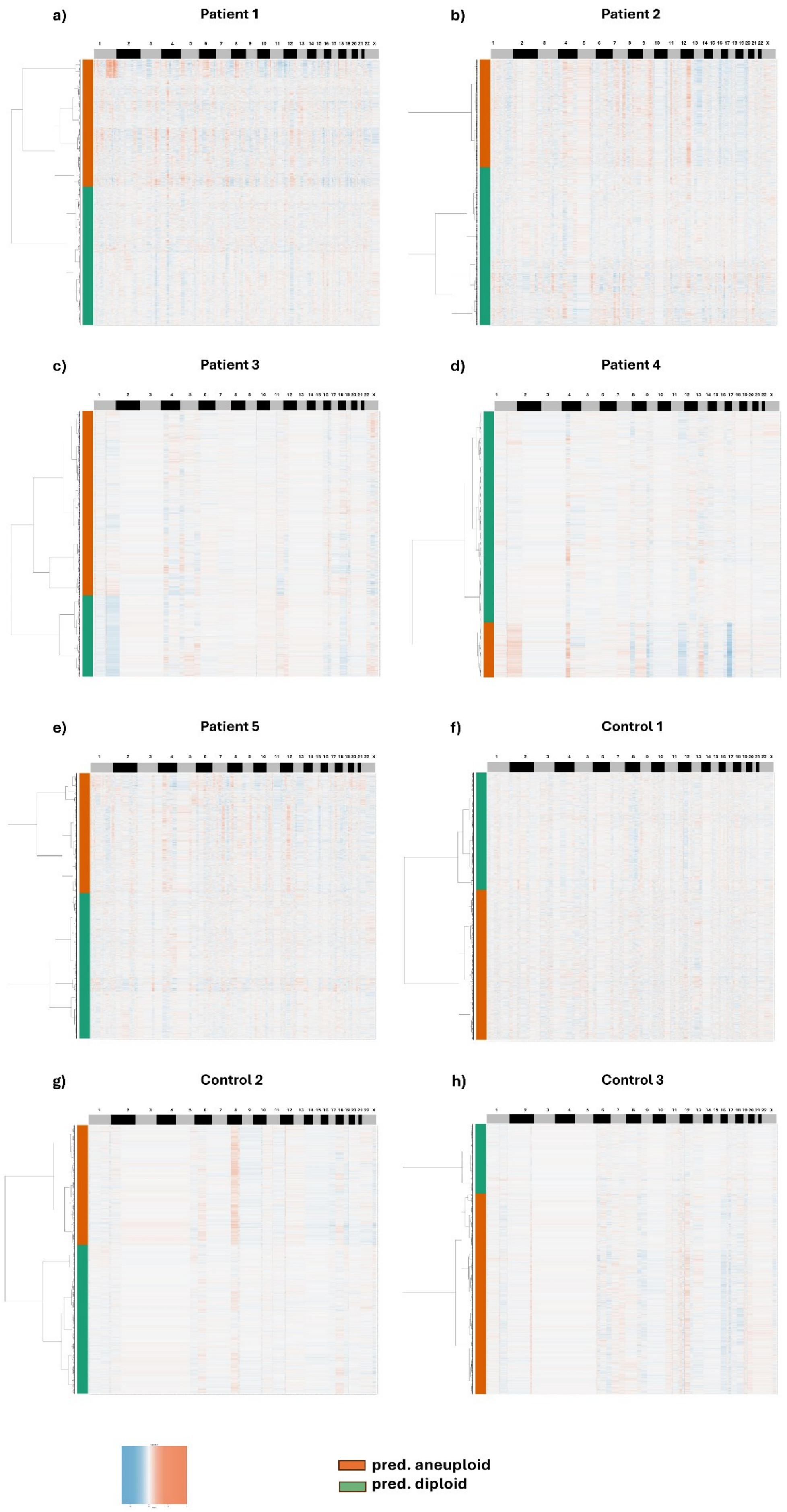
a)
Patient 1
b)
Patient 2
c)
Patient 3
d)
Patient 4
e)
Patient 5
f)
Control 1
g)
Control 2
h)
Control 3
1 2 3 4 5 6 7 8 9 10 11 12 13 14 15 16 17 18 19 20 21 22 X
pred. aneuploid
pred. diploid

Supplementary figure 3 (Figure S3):

Heatmaps of CNVs predicted in tumour cells by SCEVAN tool. The predicted tumour clusters (subclones) in the EC and control samples from the inference of CNVs in 22 chromosomes of each sample are shown: orange indicates amplifications, blue deletions and white neutral states. a) Heatmap of CNVs present in the 5 tumour subclones of patient 1. b) Heatmap of CNVs present in the 4 tumour subclones of patient 2. c) Heatmap of CNVs present in the 4 tumour subclones of patient 3. d) Heatmap of CNVs present in the 5 tumour subclones of patient 4. e) Heatmap of CNVs present in the 4 tumour subclones of patient 5. f) Heatmap of CNVs present in the 5 tumour subclones of control 1. g) Heatmap of CNVs present in the 3 tumour subclones of control 2. h) Heatmap of CNVs present in the 4 tumour subclones of control 3.

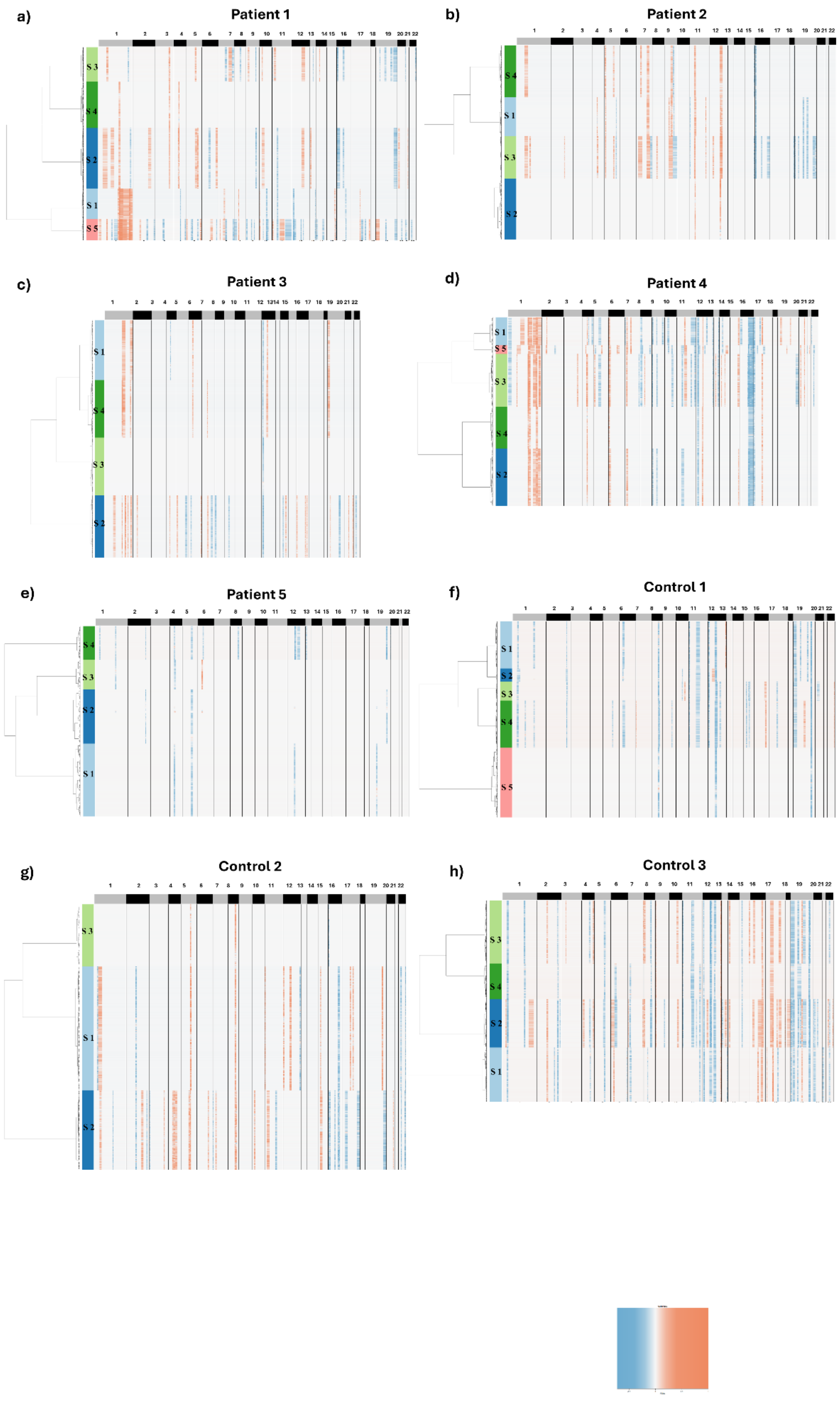
a)
Patient 1
b)
Patient 2
c)
Patient 3
d)
Patient 4
e)
Patient 5
f)
Control 1
g)
Control 2
h)
Control 3

Supplementary figure 4 (Figure S4):

Heatmaps of CNVs predicted in tumour cells by CopyKAT tool. The two predicted tumour clusters (tumour subclusters) in the EC and control samples from the inference of CNVs in 22 + X chromosomes of each sample are shown: orange indicates amplifications, blue deletions and white neutral states. Tumour subclone 1 is represented with the colour purple, while tumour subclone 2 is identified with the colour fuchsia. a) Heatmap of CNVs present in the 2 tumour populations of patient 1. b) Heatmap of CNVs present in the 2 tumour populations of patient 2. c) Heatmap of CNVs present in the 2 tumour populations of patient 3. d) Heatmap of CNVs present in the 2 tumour populations of patient 4. e) Heatmap of CNVs present in the 2 tumour populations of patient 5. f) Heatmap of CNVs present in the 2 tumour populations of control 1. g) Heatmap of CNVs present in the 2 tumour populations of control 2. h) Heatmap of CNVs present in the 2 tumour populations of control 3.

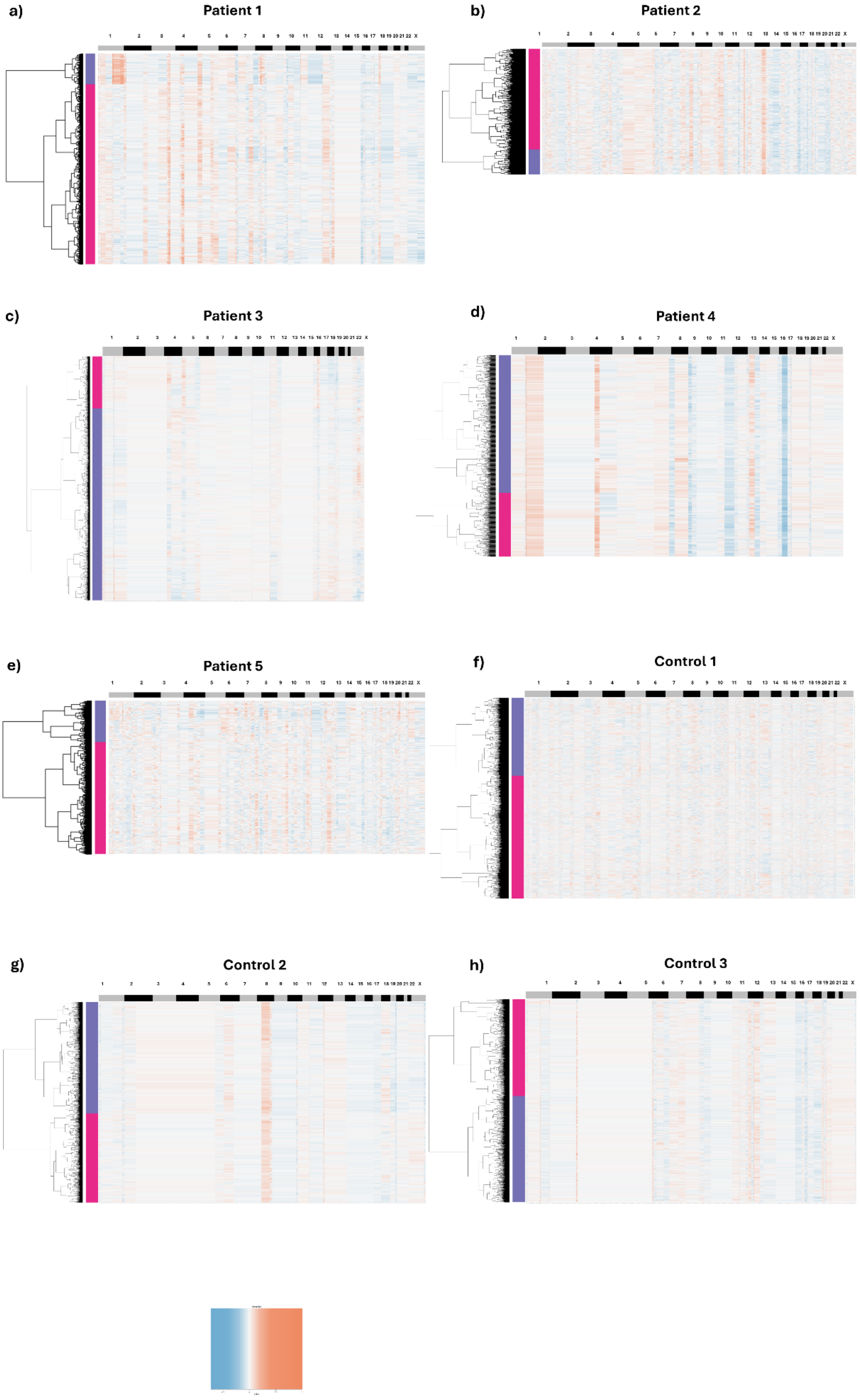

a)
Patient 1
b)
Patient 2
c)
Patient 3
d)
Patient 4
e)
Patient 5
f)
Control 1
g)
Control 2
h)
Control 3

Supplementary figure 5 (Figure S5):

Heatmaps CNVs predicted by InferCNV tool in each sample. For each sample, a heatmap of CNVs inferred in immune cells (reference cells) is shown, followed by a heatmap of CNVs inferred in other cells. In addition, the heatmap generated by InferCNV shows the inferred CNVs in 22 + X chromosomes of each sample: orange indicates amplifications, blue deletions and white neutral states.

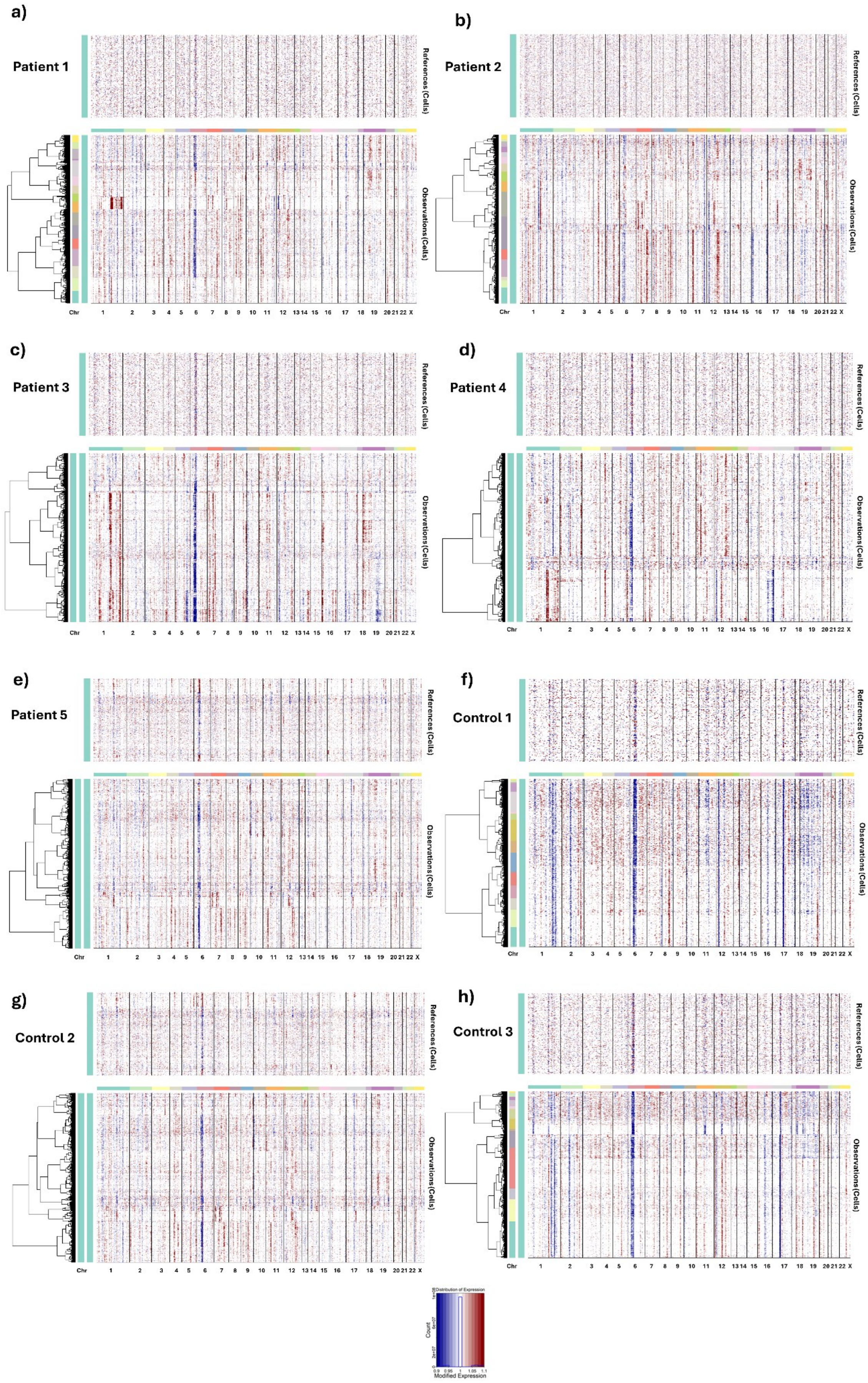
a)
Patient 1
b)
Patient 2
c)
Patient 3
d)
Patient 4
e)
Patient 5
f)
Control 1
g)
Control 2
h)
Control 3
References (Cells)
Observations (Cells)
Chr
1 2 3 4 5 6 7 8 9 10 11 12 13 14 15 16 17 18 19 20 21 22 X
Distribution of Expression
Count
Modified Expression
0.9 0.95 1 1.05 1.1

Supplementary figure 6 (Figure S6):

Beanplot of the CNV scores obtained by sciCNV tool. Each beanplot shows the CNV score of the reference cells (immune cells) and the other cells for each sample (Test). a-e) tumour samples, f-h) control samples.

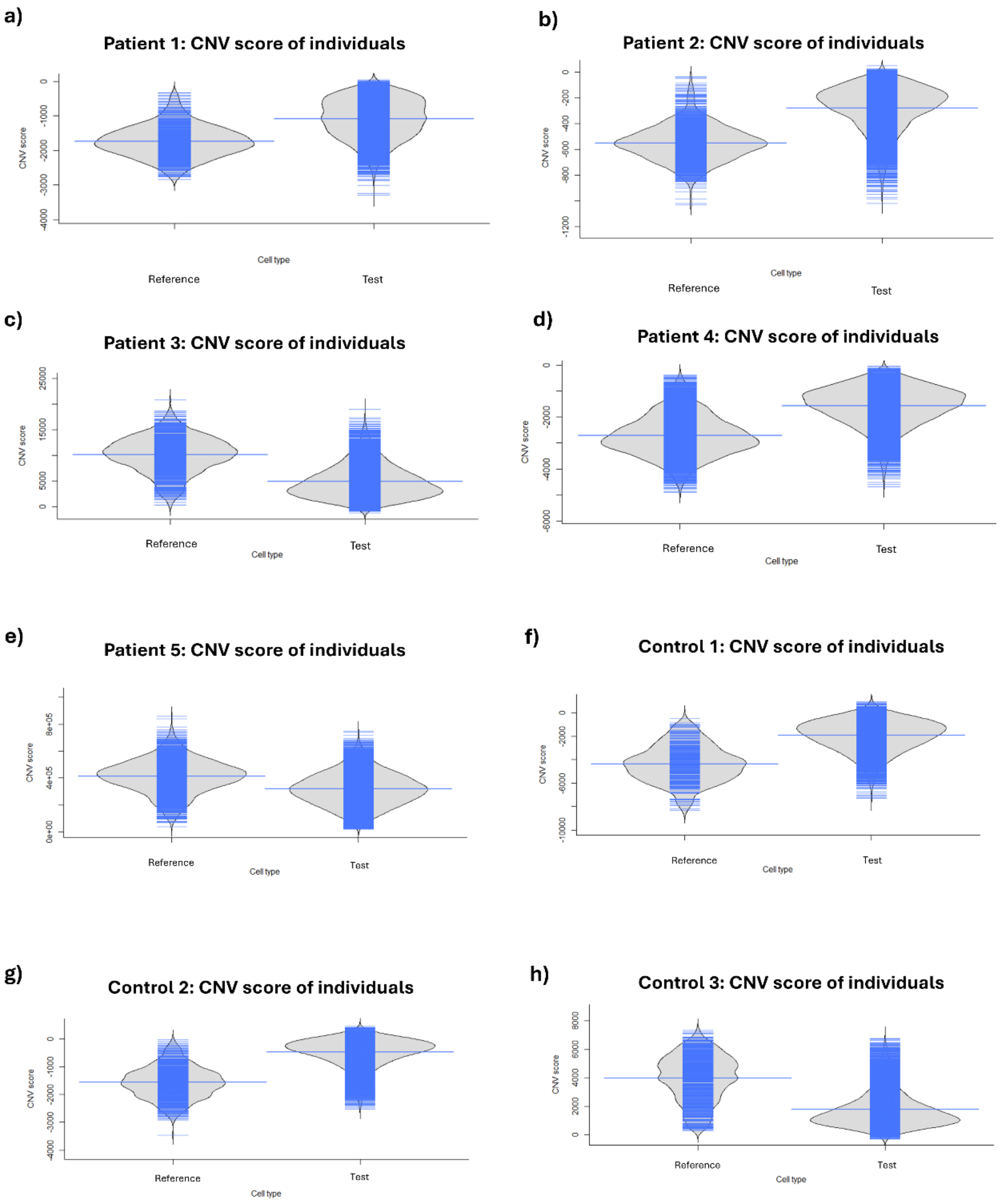

Supplement: Supplementary file 1 — Table S1: Characteristics of analysed specimens. Table S2: Overexpressed genes in Endometrial Cancer. Table S3: Comparison of CNV inference tools from scRNA‐seq. Table S4: Cell type annotation by SingleR software. Table S5: SCEVAN performance. Table S6: CopyKAT performance. Table S7: Sensitivity and specificity of the SCEVAN and CopyKAT tools. Figure S1: Heatmaps of CNVs predicted by SCEVAN tool. Figure S2: Heatmaps of CNVs predicted by CopyKAT tool. Figure S3: Heatmaps of CNVs predicted in tumour cells by SCEVAN tool. Figure S4: Heatmaps of CNVs predicted in tumour cells by CopyKAT tool. Figure S5: Heatmaps CNVs predicted by InferCNV tool in each sample. Figure S6: Beanplot of the CNV scores obtained by sciCNV tool. [file JCMM-29-e70932-s001.pdf]
